# Daily rhythms in antennal protein and olfactory sensitivity in the malaria mosquito *Anopheles gambiae*

**DOI:** 10.1038/srep02494

**Published:** 2013-08-29

**Authors:** Samuel S. C. Rund, Nicolle A. Bonar, Matthew M. Champion, John P. Ghazi, Cameron M. Houk, Matthew T. Leming, Zainulabeuddin Syed, Giles E. Duffield

**Affiliations:** 1 Department of Biological Sciences and Eck Institute for Global Health, Galvin Life Science Center, University of Notre Dame, Notre Dame, IN 46556; 2 Department of Chemistry and Biochemistry and Eck Institute for Global Health, Nieuwland Science Hall, University of Notre Dame, Notre Dame, IN 46556; 3 These authors contributed equally to this work.

## Abstract

We recently characterized 24-hr daily rhythmic patterns of gene expression in *Anopheles gambiae* mosquitoes. These include numerous odorant binding proteins (OBPs), soluble odorant carrying proteins enriched in olfactory organs. Here we demonstrate that multiple rhythmically expressed genes including OBPs and takeout proteins, involved in regulating blood feeding behavior, have corresponding rhythmic protein levels as measured by quantitative proteomics. This includes AgamOBP1, previously shown as important to *An. gambiae* odorant sensing. Further, electrophysiological investigations demonstrate time-of-day specific differences in olfactory sensitivity of antennae to major host-derived odorants. The pre-dusk/dusk peaks in OBPs and takeout gene expression correspond with peak protein abundance at night, and in turn coincide with the time of increased olfactory sensitivity to odorants requiring OBPs and times of increased blood-feeding behavior. This suggests an important role for OBPs in modulating temporal changes in odorant sensitivity, enabling the olfactory system to coordinate with the circadian niche of *An. gambiae*.

*Anopheles gambiae* is the major African malaria vector. Insecticide and drug resistance highlights the need for novel malaria control strategies. *An. gambiae* exhibits daily rhythms in physiology and behavior that include flight, mating, sugar and blood-meal feeding and oviposition^1^,^2^,^3^,^4^,^5^. Olfaction is important for detection of blood-feeding hosts, sugar feeding sources and oviposition sites^6^. Our previously reported 48 hour analysis of circadian and diel (observed under light:dark [LD] cycle conditions) rhythmic gene expression found 25 known or putative olfactory genes in female heads. These rhythmic genes include 15 OBPs, 2 other chemosensory proteins and 2 takeout (TO) homologues^7^,^8^. Many of the OBPs that had rhythmic expression under LD conditions, had reduced amplitude rhythms in constant dark, or a complete loss of rhythmicity. This highlights the contribution of the LD cycle in shaping 24-hr rhythmicity in gene expression that has been proposed before in various taxonomic groups, and has been explicitly examined in *Drosophila*^9^.

OBPs, numbering about 51 in *Anopheles gambiae* Giles (Diptera: Culicidae) (www.vectorbase.org; release VB-2013-04), are encoded by some of the most abundantly expressed antennal transcripts^10^ and are critical for the transport of hydrophobic odorant molecules across the aqueous lymph space in the antenna to the dendrite surface^10^,^11^,^12^,^13^,^14^. In mosquitoes, OBPs bind to odorant molecules, and a subset have encoding gene expression enriched in female heads/olfactory organs relative to male tissues and/or female bodies and/or are down-regulated following blood-feeding. These findings provide further evidence of OBPs role in olfaction (only the female mosquito blood-feeds)^10^,^11^,^12^,^13^,^14^,^15^,^16^. *An. gambiae* and *Culex quinquefasciatus* OBP1 are functionally characterized and are shown to bind to the host odorant indole. Moreover, the RNAi knockdown of *OBP1* results in electrophysiological insensitivity to indole in *An. gambiae*^11^ and significant reduction in *Cx. quinquefasciatus*^17^. We have recently shown that gene expression rhythms in OBPs are driven in part by both the LD cycle and the endogenous circadian clock^8^. Sensory Appendage Protein (SAPP1, AGAP008051) and A10 (homologue to *Drosophila* antennal protein 10, AGAP008055) are associated with insect sensory organs such as the antennae, bear a hydrophobic pocket, and are proposed to have chemosensory function^18^. Similar to OBPs, and structurally similar to juvenile hormone binding protein, takeout proteins have a hydrophobic pocket, and are also thought to function as ligand carrier proteins involved in chemoreception^19^. *Drosophila takeout* plays a role in linking circadian output, temporal and food information to metabolic and feeding behavioral processes^20^. Knockdown of *takeout* RNA *in vivo* reduces *An. gambiae* blood-feeding^1^, thus demonstrating an important blood-feeding role of these proteins.

Tsetse flies, *Drosophila* and cockroaches have rhythmic daily changes in olfactory sensitivity^21^,^22^,^23^, and *An. gambiae* exhibit diel and circadian rhythms of Blood-feeding behavior^1^,^5^. Previous work demonstrated the presence of rhythmic proteins in mouse liver using 2D gel-electrophoresis and mass spectrometry for protein quantification and identification, respectively^24^. Importantly, while it should not be assumed that a rhythm at the mRNA level will automatically be reflected at the protein abundance level, in many cases such coordination does occur^24^. In our study of *An. gambiae* mosquitoes we hypothesized that rhythms in olfactory gene expression would correspond with rhythms in protein abundance, and that these rhythmic profiles would correspond with time-of-day changes in detection/sensitivity to host odorants. We used quantitative multiple reaction monitoring (MRM) proteomics to measure changes in protein abundance in adult female olfactory tissues, comparing antennae and total head appendages (THA; maxillary palps, antennae and proboscis). Further, we measured olfactory responses induced by major host-derived chemicals from female adult antennae by electroantennograms (EAGs) to detect dose-dependent and time-of-day specific differences. Finally, we provide compelling behavioral evidence that confirms the rhythmic nature of flight and blood-feeding behavior in the laboratory, and that correlates strongly with our transcriptome, proteomic and physiological evidence for time-of-day specific changes in olfactory function.

## Results

### Targeted quantitative proteomics

**Figure 1:**
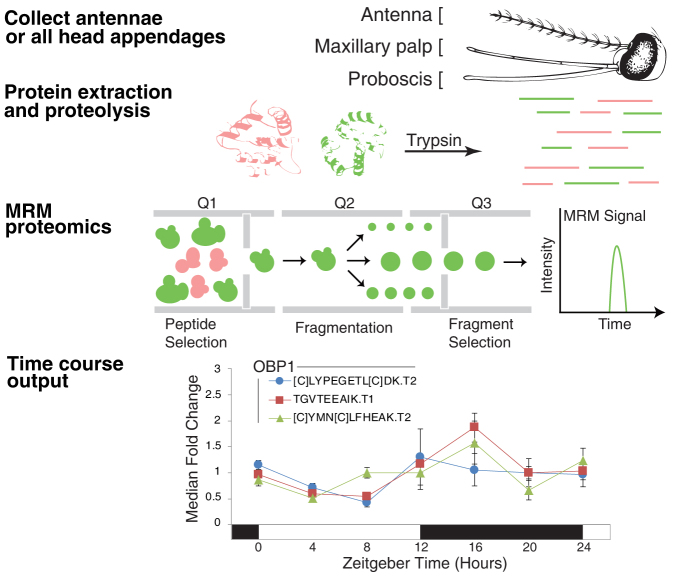
Targeted quantitative proteomics method. Our experimental method is described. Briefly, adult female heads, antennae or THAs are pulverized, proteins extracted under denaturing conditions, clarified by centrifugation, digested with trypsin, quenched and desalted. Targeted quantitative proteomics was performed using multiple reaction monitoring (MRM) on a triple quadrupole (QTrap 5500). MRM data were acquired in-triplicate or quadruplicate from samples collected every 4 hr for 24 hr. Peak areas for each peptide corresponding to the protein were integrated and the data normalized. AUC response (area under curve) was graphed for each protein *vs.* time, shown here after median transformation of the data. Note every protein had 2–3 underlying peptides used for quantification/qualification. An example of the three peptides used to quantify protein levels of OBP1 over 24 hr is shown. Targeted (quantitative MS/MS) proteomics is advantageous as it is highly specific, sensitive and linear *with respect to quantity*. In this work we demonstrate relative protein abundance rather than absolute quantities. Horizontal bars indicate day/night (white/black). See Fig. S1 for a detailed methodology and example results.

We focused our proteomics study on rhythmic olfaction genes identified in our *An. gambiae* transcriptome study^7^,^8^ in olfactory tissues. We hypothesized that rhythmic levels of these transcripts will be translated into quantifiable protein rhythms, and lead to time-of-day specific changes in olfactory sensitivity and behavior. In order to assess the quality of our protein extraction, determine which olfactory proteins were likely ‘visible' to a proteome, and generate empirical MS/MS spectra to be used for targeting, we performed data-dependent bottom-up proteomics on tryptic digests of mosquito heads, THAs and antennae (Fig. 1 and Fig. S1a). Single-dimensional nano-UHPLC MS/MS was chosen as it is identical to the level of separation analyzed by the targeted proteomics approach, thus allowing direct comparisons for verification and validation of targets. Our combined injections for bottom-up proteomics yielded a total of 1022 protein and 6157 peptide identifications at a 1% false discovery rate (Supplementary Dataset 1).

**Figure 2:**
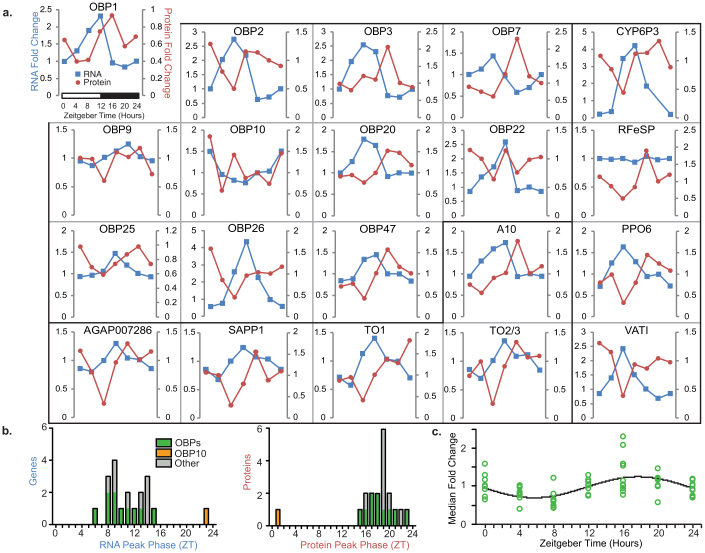
Antennal protein rhythms correspond with RNA rhythms. (a) Quantitative proteomics reveals rhythms in antennal protein abundance that correspond highly with RNA expression profiles from whole-heads. Proteins are grouped into OBPs, non-OBP chemosensory proteins (AGAP007286, SAPP1, A10, TO1, and TO2/3) and non-olfactory proteins (CYP6P3, RFeSP, PPO6, and VATI). All gene symbols as listed in VectorBase except for the following genes: RFeSP (homologue to *Drosophila* Rieske iron-sulfur protein, AGAP008955), A10 (homologue to *Drosophilla* antennal protein 10, AGAP008055), VATI (predicted V-type proton ATPase catalytic subunit I, AGAP001587) and AGAP007286 (*Ae. aegypti* OBP43 homologue). Protein abundance normalized with tubulin. See Fig. S3 for protein rhythms in the second biological replicate antennae run and THAs. (b) Histogram of peak RNA expression phases in total heads and the corresponding peak antennal protein levels as determined by cosinor analysis from genes/proteins in panel A. (c) Cosinor analysis of antennae OBP protein levels from panel A (p < 0.001; acrophase ZT17.7); note OBP10 excluded as it has antiphasic expression compared to the other OBPs.

In order to determine if proteins identified by qualitative proteomics had rhythms specific to sensory tissues, we utilized targeted quantitative MRM proteomics to both identify and determine concentration in antennae and THAs^25^,^26^,^27^,^28^,^29^,^30^. Biological material was collected every 4 hr for 24 hr from adult females maintained under strict LD cycle conditions (12 hr light: 12 hr dark including 1 hr dawn/dusk transitions). In antennae and THAs, we were able to defensibly quantify 21 of 25 targeted proteins, respectively, including 11 OBPs and 5 other putative chemosensory proteins (Fig. 2, Fig. S3). The profiles of protein abundance for these proteins were found to be rhythmic. Detected OBPs include OBP1 (AGAP003309), known to bind indole and DEET^11^,^15^, and OBP20 (AGAP005208), known to bind DEET, 6-methyl-5-heptone-2-one and indole^14^. Detected and rhythmic in our analysis, OBP1, 2 (AGAP003306), 3 (AGAP001409), 20, 22 (AGAP010409), 25 (AGAP012320), 26 (AGAP012321) and 47 (AGAP007287) are reported to have gene expression enriched in female heads/olfactory organs relative to male tissues and/or female bodies^10^,^12^,^13^. A recent RNA-seq experiment also revealed that many of these *OBP* genes are down-regulated in antennae following blood-feeding and as the mosquito transitions to oviposition behavior^16^. These studies provide further evidence that these OBPs in particular are potentially involved in olfactory host-seeking behavior in females. Two other chemosensory proteins, SAPP1 and A10, were also found to be rhythmic. Additionally, the takeout proteins TO1 (AGAP004263) and TO2/3 (AGAP012703/AGAP004262), which have been implicated in chemoreception and in blood-feeding behavior^1^,^19^, were detected and rhythmic in antennae and THAs. Many protein rhythms were robust, with 3.2, 8.4, 4.5 and 5.3 peak-to-trough fold changes observed for OBP1, OBP26, TO1 and SAPP1 antennae protein abundance, respectively.

In general, olfactory proteins were at their highest abundance at Zeitgeber time (ZT) 16 and lowest between ZT4 and ZT8 (ZT12, lights off; ZT0, end of dawn transition; Fig. 2 and Fig. S3). Protein profiles were compared with corresponding time-specific mRNA profiles, reanalyzed in 24 hr format from our 48 hr time course microarray analysis^7^, and subjected to identical cosinor analysis to determine peak phase. Most of the proteins had a rhythmic phase lagging their corresponding RNA expression rhythm. With the exception of OBP10 (AGAP001189), which had antiphasic RNA and protein levels, OBP RNA rhythms peaked at ZT10.1 ± 0.9 (hr ± S.E.M.) and protein rhythms at ZT16.8 ± 0.5 (measured by cosinor analysis), resulting in a peak OBP RNA to protein lag of 6.9 hr ± 0.7 hr (Fig. 2b,c). This common peak in OBPs was consistent across biological replicates and between antennae and THAs (cosinor analysis peak in antennae replicate 1, ZT18.4 ± 0.7 [group analysis, ZT17.7]; replicate 2, ZT16.9 ± 0.8 [ZT16.8]; and in THAs, 15.0 ± 0.9 [ZT14.9]; Fig. 2b–c and Fig. S3b–f). Such a large phase lag between RNA and protein rhythms has been observed previously^24^,^31^, *e.g.* in mouse liver the RNA-protein lag of several clock components and clock controlled genes, such as *per1*, *per2*, *Id2* and *Sdh1*, is 6–9 hr^24^,^31^,^32^. The lag could be explained by the additional action of post-transcriptional^33^ and post-translational processes (*e.g.* protein phosphorylation and turnover), and our previously published gene expression analyses suggest that these mechanisms are in fact rhythmically regulated in *An. gambiae*^7^.

As part of our proteomics analysis we additionally targeted three proteins with rhythmic mRNA levels^7^,^8^ that are specifically involved in metabolic detoxification (CYP6P3, AGAP002865), immunity/cuticle pigmentation (PPO6, AGAP004977) and vesicular type H^+^ ATPase (VATPase) activity (VATI, AGAP001587): In all cases, protein levels were found to be rhythmic (Fig. 2, Fig. S3), and therefore suggests functional changes. As CYP6P3 metabolizes pyrethroids, such rhythmicity may confer time-of-day specific changes in insecticide resistance; PPO6 is a key enzyme involved in the melanization process, an important immune sequestering process; and changes in VATPase activity, amongst several things, contributes to synapse function and neurotransmitter recycling, see^7^,^8^. It is plausible that rhythmicity in this process might contribute additionally to the temporal changes observed in olfactory sensitivity (see electrophysiological analysis, below). In THAs, in addition to proteins reliably detected in antennae, we also defensibly detected and profiled an additional three non-olfactory proteins which were rhythmic at the transcript level^7^ (Fig. S3d): The citric acid cycle enzyme isocitrate dehydrogenase (IDH; AGAP006660), the glutathione-S-transferase metabolic detoxification protein GSTD7 (AGAP004163), and Actin5C (AGAP000651). Note that the *Drosophila* Act5C gene promoter has been used frequently for transgenic mosquito experiments.

Not everything in our preparations had a protein profile with a peak during the early to mid-night phase (ZT12-20). In fact we observed profiles suggesting rhythmic peaks earlier in the 24 hr day; e.g. in THAs GSTD7 has a cosinor peak phase estimate of ZT9.4 and a peak expression measured at ZT4. There were several patterns of protein and RNA where this phase coordination was notably different: In both antennae and THAs RFeSP had a constitutively expressed RNA profile but rhythmic protein levels, while in THAs, GSTE3 (AGAP009197) displayed the opposite phenomenon, with rhythmic mRNA levels but corresponding constitutive protein levels. GSTE3 has been implicated in insecticidal detoxification^34^. Furthermore, in THAs the Actin5C protein profile was antiphasic to its encoding mRNA (cosinor peak phase estimate ZT3.0 and ZT16.7 for RNA and protein, respectively). Such a distinct absence of phase coordination between RNA and protein has been reported before^24^,^33^. For example, Reddy *et al*. similarly found half of cycling mouse liver proteins lacked a corresponding rhythmic mRNA; a phenomenon they attributed to post-transcriptional processes^24^,^33^. However, considering that our mRNA profiling was performed in total heads and protein analysis from specific olfactory tissues, we cannot exclude the possibility of tissue-specific mRNA rhythms that might correspond better with observed protein results.

**Figure 3:**
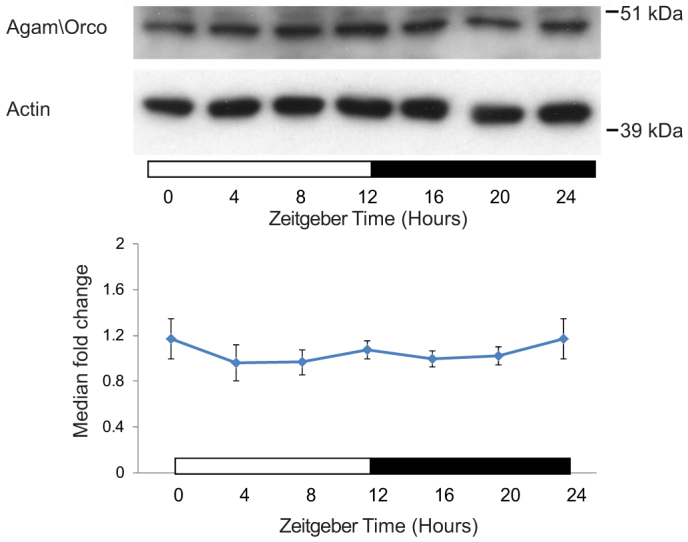
Agam\Orco immunoblot analysis. Protein levels across 24 hr (n = 5 biological replicates). Representative blots are shown (top). Levels of Agam\Orco were determined by normalizing Agam\Orco blots to their respective β-actin signal. Blot images shown have been cropped to highlight the relevant band. Internal normalization was conducted by making the median value of each time course equal to 1.0. One-way ANOVA was performed to determine for time-of-day differences (n.s.). The data from Zeitgeber time 0/24 are double plotted. We also observed a second prominent band at ~100 kDa, which we predict is an Agam\Orco heterodimer; and this was also constitutively expressed across the 24 hr. Horizontal bars indicate day/night (white/black).

Using proteomics we could not defensibly detect the obligatory co-receptor required for all odorant receptor (OR) function, Odorant Receptor Coreceptor Agam\Orco, also known as Or7 (AGAP002560)^35^,^36^. RNA levels of *Agam\Orco* are rhythmic, peaking at ZT10 (prior to dusk and the onset of nocturnal behavioral activities involving olfaction)^1^,^2^,^3^,^4^,^5^,^7^. We previously hypothesized that rhythmic Agam\Orco protein levels could serve as a mechanism for rhythmic control of olfactory sensitivity^7^. However, immunoblot analysis revealed constitutive Agam\Orco levels (i.e. no significant differences in protein abundance were detected by Kruskal-Wallis ANOVA; cosinor analysis, n.s.; Fig. 3), indicating Agam\Orco is unlikely to gate rhythmic olfactory sensitivity. Note that we were unable to detect any odorant-specific ORs in our microarray analysis^7^ or proteomic analyses, presumably due to their low abundance^16^.

### Electrophysiological analysis

**Figure 4:**
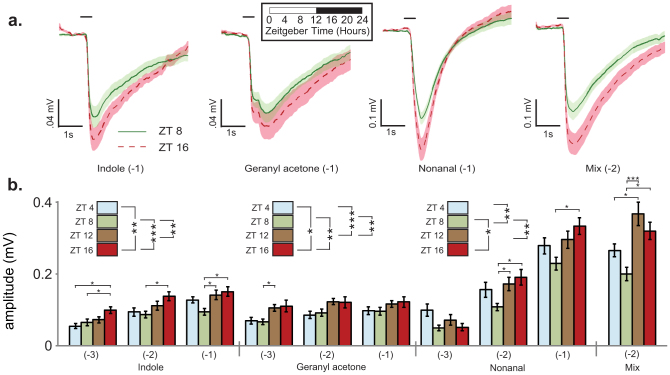
Time-of-day changes in mosquito olfactory sensitivity to hydrophobic host constituents. (a) Electrophysiological analysis of adult female mosquito antennae at different times of the day. For each stimulus, the mean ± S.E.M. trace at ZT8 (afternoon) and ZT16 (night) are graphed. The black bar at 1.0 s indicates the onset of 0.5 s stimulus delivery. See Fig. S4 for dose-dependent traces. (b) EAG responses at different times of day and at different stimulus concentrations. Different stimulus compounds are indicated on the bottom horizonatal axis. Indole (two way ANOVA: Effect of time (t), F_2,182_ = 26.7, p < 0.001; effect of concentration (c), F_3,182_ = 9.7, p < 0.001; interaction (i), F_6,182_ = 0.7, n.s.); Nonanal (two way ANOVA: t, F_3,192_ = 7.2, p < 0.001; c, F_2,192_ = 155.6, p < 0.001; i, F_6,192_ = 1.8, n.s.); geranyl acetone (2 way ANOVA: t, F_3,183_ = 8.0, p < 0.001; c, F_2,183_ = 4.1, p < 0.05; i, F_6,183_ = 0.3, n.s.). Significant results of Tukey *post hoc* tests at each time-of-day (at a given concentration) are indicated above bar graphs. Overall effect of time-of-day indicated in panel legends. Significant time-of-day differences were also detected within mix (−2) using Dunn's method *post hoc* tests (Kruskal-Wallis ANOVA: H = 17.0, df = 3, p < 0.001). n = 12–24 recordings per group. Bar charts represent mean ± S.E.M values. *p < 0.05, **p < 0.01, ***p < 0.001.

We next determined if the temporal rhythms described in protein levels correspond with olfactory sensitivity. We used electroantennogram (EAG) analysis to measure the olfactory responses induced by host-derived odorant chemicals. At ZT4 (morning), ZT8 (afternoon), ZT12 (dusk) and ZT16 (night), mosquito antennae were challenged with major host-derived odorant chemicals (nonanal, indole, geranyl acetone, and a *mixture* of hexanoic acid, geranyl acetone, nonanal, indole, 3-methylindole and *p*-cresol). As several of the odorants are hydrophobic, it is expected that OBPs will be necessary for the mosquito to detect these stimuli. Since OBPs are rhythmic in protein abundance, we predicted rhythmic sensitivity in detection of these compounds. Indeed, we found time-of-day specific olfactory responses to all four stimuli, with sensitivity peaking at ZT16 (night) and least at ZT4 or ZT8 (day) (Fig. 4a–b). In all cases, the time-of-day specific differences in EAG amplitudes were comparable to the differences observed due to 10-fold changes in odorant concentration (mean peak-to-nadir difference in each time series: Geranyl acetone 44%, indole 50% and nonanal 61%; mean increase in response with each 10× increase in odorant concentration: Geranyl acetone 15%, indole 67%, and nonanal 81%). It is therefore plausible that the ≤8.4-fold rhythmic changes observed in OBP abundance (antennae mean ± SEM fold-change, 3.4 ± 0.4; THAs, 3.2 ± 0.6) could account for such differences in EAG responses. This is especially so given that in some cases multiple OBPs can bind a single odorant, and vice versa^11^,^14^,^15^,^17^. These findings are broadly consistent with our observed OBP protein rhythms (Fig. 2, Fig. S3) and mosquito behaviors described here (Fig. 6) and by others^1^,^5^. Specifically, protein expression and olfactory sensitivity were also lowest at ZT4-ZT8 and highest at ~ZT16, and blood-feeding and flight behaviors were higher at night than during the daytime. These data are particularly interesting for indole as there is significant evidence that OBP1 contributes to antennal sensing of this odorant^11^,^17^. OBP1 protein rhythm has a nadir at ZT4-ZT8 and a peak at ZT16 (Fig. 1, Fig. 2), which corresponds with the lowest and highest sensitivity to indole in our electrophysiological measurements.

**Figure 5:**
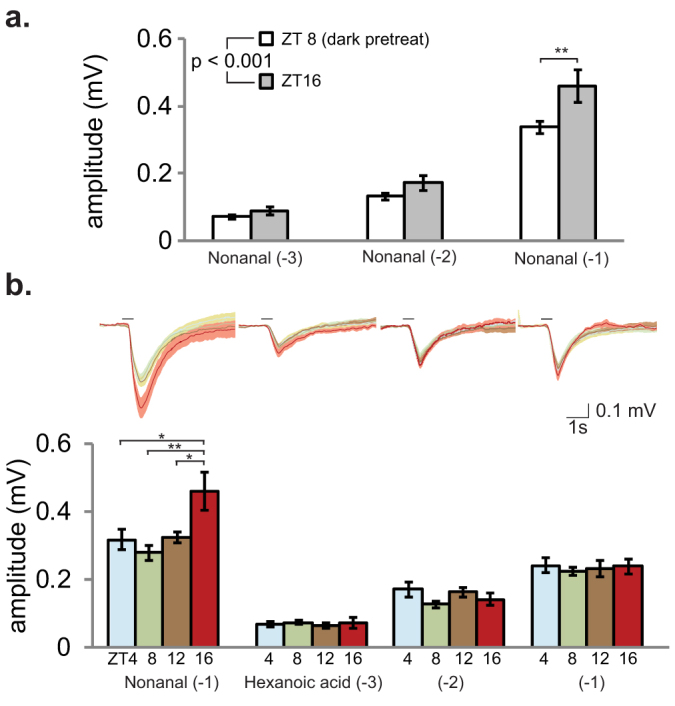
Temporal differences to a hydrophobic odorant are maintained in dark treated mosquitoes, but there are no time-of-day specific changes to hexanoic acid. (a) Time-of-day differences were retained between mosquitoes at ZT16 and mosquitoes at ZT8 pretreated with dark for 4 hr (two way ANOVA: t, F_1,36_ = 15.4, p < 0.001; c, F_2,36_ = 192.7, p < 0.001; i, F_2,36_ = 1.9, n.s.). For nonanal (−1), Tukey *post hoc* tests also revealed a difference between ZT8 and ZT16 (p < 0.05). n = 5–9 recordings per group. (b) Mosquito EAG responses to hexanoic acid were concentration dependent, but not time-of-day dependent (two way ANOVA: t, F_3,77_ = 0.8, n.s.; c, F_2,77_ = 106.0, p < 0.001; i, F_6,77_ = 0.6, n.s.). Traces represent, the mean ± S.E.M. EAG responses at all three concentrations and all four tested time points. n = 6–8 recordings per group. The nonanal control showed expected time-of-day specific changes in sensitivity (ANOVA: F_3,26_ = 5.9, p < 0.01) and Tukey *post hoc* test results are shown. Bar charts represent mean ± S.E.M values. *p < 0.05, **p < 0.01.

Next, we looked at olfactory responses to nonanal (host odorant that elicited highest EAG amplitude response from our panel) at ZT16 and at ZT8 from normally reared mosquitoes, but tested under identical lighting conditions. In order to control for any acute effects of light or dark on influencing the odorant response, the ZT8 group was pretreated with 4 hr of darkness prior to EAG recording, and both ZT8 and ZT16 animals were tested under red light (Fig. 5a). It is plausible that light could influence olfactory function, either acting via the compound eye, or acting directly on the antennae via the blue light photoreceptor cryptochrome 1 (CRY1, AGAP001958), which is likely to be expressed locally in chemosensory organs of the mosquito^7^,^37^,^38^. Consistent with the experiments conducted under normal LD cycle conditions (Fig. 4a–b), this analysis revealed both a concentration dependent and a time-of-day effect: Mosquitoes at ZT16 retained a higher EAG sensitivity than dark-treated ZT8 mosquitoes. This indicates that reduced sensitivity at ZT8 is not an acute effect of light on the function of the antennae, but in fact a reflection of an underlying circadian clock and/or diel regulated mechanism (driven by the environmental 24 hr LD cycle)^8^. Further, 24 hr rhythmic changes in electrophysiological sensitivity persist in constant dark (DD) conditions in *Drosophila* and cockroaches^21^,^23^, and are driven by peripheral clocks located in the *Drosophila* antennae themselves^39^. Therefore, it is likely that the observed time-of-day changes in EAG sensitivity in *An. gambiae* are driven not by an acute effect of light or darkness, i.e. a masked response, but by an underlying clock and/or diel mechanism. This corresponds with our previous work that suggests that OBP gene expression is dependent upon both mechanisms working in concert^8^.

Finally, we tested the time-of-day specific olfactory sensitivity of mosquitoes to hexanoic acid, a hydrophilic host odor constituent that does not require OBPs for detection. Mosquito olfactory responses were measured with increasing doses of hexanoic acid, and compared to nonanal, which served as a positive control. As expected, responses to the nonanal control exhibited time-of-day sensitivity, peaking at ZT16, while responses to hexanoic acid did not change (two way ANOVA; Tukey *post hoc* tests, n.s.; Fig. 5b). Acid responses were however dose dependent (all concentrations are significantly different from each other; Tukey *post hoc* tests, p < 0.001). Earlier investigations of female *Culex pipiens* mosquitoes also did not reveal a time-of-day specific change in electrophysiological sensitivity to lactic acid^40^, which presumably does not require an OBP for detection.

### Flight and blood-feeding behavior rhythms

**Figure 6:**
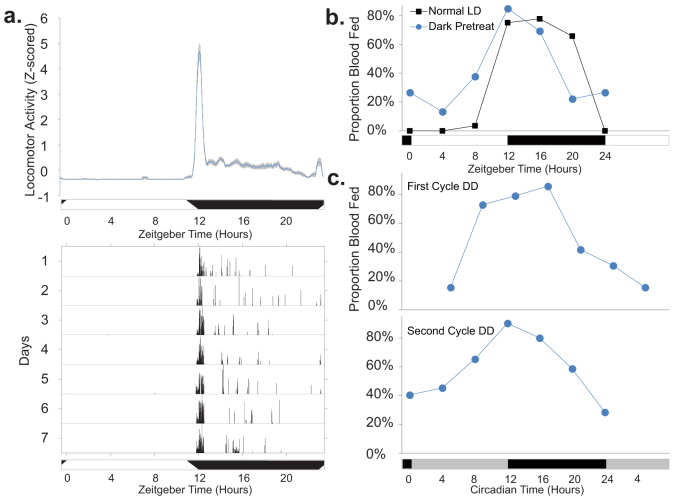
Flight behavior and blood-feeding rhythms. (a) Flight behaviors of host-seeking adult female *An. gambiae* mosquitoes. (Top) Mean ± S.E.M of Z-scored 30 min running averages of locomotor/flight activity of 19 mosquitoes monitored over 7 days. See Rund *et al.* 2012 for an analysis of male and virgin female flight behavior profiles^3^. (Bottom) Representative actogram of an individual female mosquito maintained under 12:12 LD conditions (with 1 hr dawn and dusk transitions). Each horizontal line represents a 24 hr period, and vertical bars represent time specific incidences of movement activity across an infrared beam. Numbers on the left indicate the number of days in the study. (b) Profile of the proportion of *An. gambiae* host-seeking mosquitoes blood-feeding during a 6 min exposure to a human arm under normal LD conditions (or with a 15 min of dark pretreatment). All exposures occurred in the dark. (c) Host-seeking *An. gambiae* blood-feeding in the first or second cycle of DD conditions. Horizontal bars indicate day/night (white/black) or subjective day/night (gray/black).

Having established a clear time-of-the day modulation of abundant olfactory components in adult female *An. gambiae* using transcriptomic and proteomic approaches and sensory physiological evidence, we investigated if the *An. gambiae* strain from our laboratory maintained diel flight and blood-feeding behaviors typical of mosquitoes observed in the wild. Flight activity of host-seeking mosquitoes was monitored using an infra-red beam break system^3^, and our data revealed a clear onset of mosquito behavior at the beginning of the night (Fig. 6a), a finding consistent with laboratory and field observations^5^,^41^. Blood-feeding behavior has previously been reported to be under circadian regulation in laboratory conditions using a membrane feeder^1^ and to be nocturnal in the field^42^. In the current study, blood-feeding preference was measured in our laboratory by exposing a human arm every 4 hr to individual cages of host-seeking females. After 6 min exposures, mosquitoes were visually examined for the presence of blood in their abdomens. We found that under diel conditions, blood-feeding was almost exclusively restricted to the night phase, with peak biting occurring in the early night (Fig. 6b), and coincident with timing of flight activity (Fig. 6a). The rhythm in blood feeding preference was found to persist for 2 days under DD conditions (Fig. 6c), demonstrating that this temporal behavior is driven by the endogenous circadian clock. We note the peak in blood-feeding preference (ZT12-16) is coincident with the peak time of olfactory sensitivity as measured by EAG. It is also coincident with the peak in abundance of olfactory proteins such as the takeout proteins, known to be involved in regulating feeding behavior, and OBPs.

## Discussion

This work provides additional compelling evidence of the important role of daily rhythms in the sensory biology of *An. gambiae*, the primary malaria vector. It has been hypothesized that expression dynamics of chemosensory genes may result in overt behavioral phenotypes^16^. Here, we identify the pre-dusk/dusk peak in expression of OBPs detected by microarray analysis^7^,^8^ (genomics) corresponds with peak protein abundance (proteomics), and is coincident with the time of increased olfactory sensitivity to host odorants thought to require OBPs for detection (electrophysiology), and times of increased biting (blood-feeding) behavior. We also find that neither olfactory sensitivity to an odorant that does not require OBPs for detection (i.e. hexanoic acid), nor protein abundance of the olfactory co-receptor OR, Agam\Orco, changes across the 24 hr day. Rhythmic expression of OBPs peak at the time of highest olfactory sensitivity to hydrophobic odorants (~ZT16). Therefore, these results strongly suggest that rhythms in OBPs contribute to the changes observed in sensory and behavioral function. This is not surprising given that the knockdown of a single OBP, OBP1, results in a significant decrease in the sensitivity of major chemostimuli, including indole, in *An. gambiae*^11^ and in *Cx. quinquefasciatus*^17^.

Though our data provides compelling evidence suggesting OBPs and other chemosensory proteins modulate olfactory sensitivity and behavioral outputs, we are aware that our quantitative transcriptome and proteome analyses did not detect another major chemosensory family, the ORs. Recent investigations have demonstrated a modulation of OR and OBP gene expression based on *An. gambiae* physiology, and how such differences are coincident with a switch from host-seeking to oviposition behaviors. However, these changes in OR expression were modest as compared to the OBPs^16^. Our own investigation into the role of ORs was inconclusive: The most abundant OR, Agam\Orco, did show a weak RNA rhythm in our microarray analysis^7^, but we found no evidence of rhythmicity in protein abundance. Furthermore, additional downstream signal transduction components, as previously suggested^39^, or rhythmic trafficking of olfactory components^43^ may also modulate olfactory responses. The rhythmic blood-feeding responses described here are likely driven by several factors, including time-of-day changes in sensitivity to host odorants (which may be driven by rhythmic OBP levels we describe here); rhythmic responses to CO_2_, as reported in the hematophagous *Triatoma infestans*^44^; and rhythmic protein abundance of behavioral/chemosensory factors, such as the takeout proteins TO1 and TO2/3, that we demonstrate to be rhythmic. Working alone or in concert with these above mentioned regulatory factors, it still remains a compelling hypothesis that rhythms observed in sensory organ OBP and takeout protein abundance could confer these dramatic time-of-day specific changes in odorant sensitivity and feeding behavior. The coincident times of peak protein abundance, olfactory sensitivity and behavior reflect the extraordinarily fine-tuned control of mosquito physiology, with OBP and other chemosensory protein abundance and high olfactory sensitivity up-regulated when needed (at night) and down-regulated when not required (daytime).

Our work highlights the important role of circadian/diel biology in the mosquito. Improved understanding of biological timing at the molecular level that underlies key physiological aspects of *An. gambiae* may prove to be important for the successful implementation of existing or novel control methods and future experimental design. Further, greater understanding of the rhythmic nature of blood-feeding is important. There is growing evidence that the use of insecticide-impregnated bed nets is acting as a selective pressure, potentially modifying both age and genetic composition of the mosquito population^45^. Thus, selection for mosquitoes that host-seek at times of the day when humans are not protected under bed nets may be occurring.

## Methods

### Biological material

*An. gambiae* Pimperena S form mosquitoes [MRA-861] were maintained at 85% relative humidity and 27 ± 1°C on a 12 hr/12 hr LD cycle (11 hr full light, 11 hr darkness (0 lux) and 1 hr dawn and 1 hr dusk transitions). Time of day is reported in 24 hr Zeitgeber time (ZT) with ZT12 defined as time of lights off under the LD cycle, ZT0 defined as end of the dawn transition and ZT11 is defined as the start of the dusk transition. Access to 20% high fructose corn syrup (HFCS) was provided *ad libitum*. For proteomics, three separate collections of ~30 mated but not blood-fed adult female 4–7 d old mosquitoes (i.e. host-seeking) were harvested on dry ice every 4 hr for 24 hr, and 40 antennae or 20 total head appendages (THAs; maxillary palps, antennae and proboscises) were pooled per time point.

### Proteomics

#### Protein extraction and preparation

Mosquito antennae and THAs were processed by cryogenic freezing with liquid N_2_ followed by manual pulverization with a 1.5 ml-tube pestle (USA Scientific, Ocala, FL) for 3 rounds of 15–30 s. Samples were then extracted with a mixture of equal volumes of 2,2,2, trifluoro-ethanol, 50 mM ammonium bicarbonate pH 8.0 (Fluka, Sigma-Adlrich, St. Louis, MO) supplemented with 1 mM EDTA and 1 mM PMSF (Sigma, Sigma-Aldrich)^46^. Extractions were performed for 15 min with shaking, and samples were clarified by centrifugation. Protein samples were precipitated by addition of 6-volumes of ice-cold acetone at −20°C for 1 hr, centrifuged, decanted and dried. Protein pellets were re-suspended, digested using 2,2,2 trifluoroethanol^46^, re-suspended, reduced with 25 mM DTT (Sigma) for 1 hr at 56°C, then alkylated at room temp for 20 min with 35 mM iodoacetamide (Sigma). Samples were digested with addition of 500 ng of sequencing grade trypsin (Promega, Madison, WI) for 2 hr at 37°C with mild shaking, with an additional 1 μg added for overnight digestion; quenched by addition of trifluoroacetic acid (Optima, Thermo Fisher Scientific, Waltham, WA); lyophilized in a speed-vac concentrator; re-suspended in 0.1% TFA; and desalted with a C18 Ziptip (EMD Millipore, Billerica, MA) per manufacturer's instructions.

#### Qualitative LC/MS/MS

The protein-digest extracts of *An. gambiae* antennae, THAs and heads were subjected to bottom-up LC/MS/MS analysis. Briefly, duplicate injections of antennae, THAs or head digests were separated on a 100 μm × 100 mm C18 BEH column (Waters, Milford, MA) over a 90 min gradient from 2–35% acetonitrile 0.1% formic acid (FA) (Burdick and Jackson, Honeywell, Morristown, NJ). MS and MS/MS data were acquired on an LTQ Velos Orbitrap instrument (Thermo Fisher Scientific) using a TOP8 method. Peak lists (mgf) were generated using RAW2MSM and subjected to database searches against the current version of the *An. gambiae* genome sequence from VectorBase (release VB-2013-04). The Paragon algorithm within Protein Pilot (ABSciex, Framingham, MA) was used for search and false-discovery rates were calculated with the PSPEP tool using the method of Elias *et al*^47^. The results of the combined analysis are available in Supplementary Dataset 1, and represent one of the largest collections of sensory protein identifications in mosquitoes to-date. The combined injections for bottom-up proteomics yielded a total of 1022 protein and 6157 peptide identifications at a 1% false discovery rate (Supplementary Dataset 1) that compares in magnitude to the ~6,000 *An. gambiae* proteins comprehensively identified in Chaerkady *et al*^48^. No odorant receptors (ORs) were defensibly identified at this stage after searching the bottom up generated development data. OR protein abundance was presumably below the detection limits of the experimental design; or the preparation used excluded their extraction. Note that OBP3 (AGAP001409) and OBP10 (AGAP001189) were only defensibly detected in the antennae in the second biological replicate time course: For the purposes of presentation, these data are shown with the first replicate time course (Fig. 2). Raw protein identification data, including data from which the MRMs were derived, have been deposited into the PeptideAtlas: http://www.peptideatlas.org/PASS/PASS00300.

#### MRM development, validation and acquisition

MRM transitions were determined largely from the empirical MS/MS data obtained from the bottom-up proteomics approach. Our analytical work was comparable to that employed in our previous MRM efforts^27^,^28^,^49^ and in agreement with the guidelines described by the Aebersold group^29^,^50^. A combination of empirical (MS/MS), MRM-initiated full-scan sequencing and published spectra were the source for the MRM peptide transitions. Specific transition development was performed using Skyline (MacCoss Lab) and MRMPilot (ABSciex) combined with manual refinement. An example of each type of peptide optimization is shown in Fig. S1b–g; including MRM-initiated full scan sequence confirmation performed as previously demonstrated^51^.

Briefly, 4–9 transitions were chosen for each peptide used to describe a protein of interest for as many as 4-peptides per protein (several proteins only yielded one reproducibly detectable peptide for quantification). Transition validation and determination was made according to our previously published work and published sample validation methods^29^. Transitions were compiled and reduced to quantifier and qualifier transition lists once optimized and validated by repeated injection, retention time (RT) prediction, RT cross-validation between disparate sample types (e.g. antennae, THAs and heads) and full-scan MS/MS confirmation from the bottom-up data and MRM-triggered MS/MS analysis. The final antennae list contained 214 transitions (Supplementary Dataset 2) with individual dwell times that were for the most part inversely proportionate to the observed signal from the development data. This was performed to decrease the cycle-time thereby improving the sampling rate of the data (Supplementary Dataset 1). These were subsequently re-confirmed and tested using bulk mosquito-head extracts as a complex and complete sample stress-test. For each peptide analyzed, 3–4 transitions were chosen, similar to what we and others have observed^28^,^30^,^50^. Tubulins, due to their 24 hr constitutive RNA and protein expression, are frequently used in insect circadian experiments as an immunoblot loading control, including butterfly antennae^38^,^52^. β-tubulin (AGAP010929) was used as an internal standard for normalizing protein levels across the time courses: Peptide transition EIVHIQAGQ[C]GNQIGAK was used for antennae and YLTVAAVFR for THA normalization.

MRM data were acquired on a QTrap 5500 (ABSciex) running in triple-quadrupole or hybrid ion-trap mode as previously published^28^. MIDAS experiments (MRM-triggered Data-Dependent) were acquired using the hybrid triple quadrupole-ion trap transition mode. 2 μl of the samples as prepared earlier from each time point were injected in-triplicate onto a 100 mm × 100 μm C18BEH column (Waters) running at 600 nl/min. A 90 min gradient from 2–35% acetonitrile-water (0.1% FA) was used with pre-run solvent blanks injected. Mid-run washes and quality control peptide injections were performed every 12 injections. Including tuning, approximately 100 LC/MS/MS (MRM) acquisitions were acquired. An example chromatogram of a complete MRM trace, for THAs collected at ZT16, is shown in Fig. S1d. Overall peak retention and reproducibility were excellent. Some runs exhibited a slight < 1 min (1–2%) systematic drift in retention-time (RT) throughout the >100 hr of acquisition, but this was readily corrected for with integration parameters. Most runs exhibited excellent stability. For example, RT stability for the β-tubulin peptide. FPGQLNADLR. (2y8) was 0.31%CV for all injections of antennae samples. This is typical of peptides observed in the antennae.

#### Peak Processing

Peak areas were integrated using MultiQuant (ABSciex) as described in Li *et al*^49^. Briefly, 30 s RT windows were used, and a 3-point Gaussian smooth was applied to all transitions. Peak area integrations of the quantifier MRM transition were converted to area ratio/area response by dividing by the peak area of the associated internal standard peptides from β-tubulin. Additionally, identical numbers of antennae or THAs were processed at each time point ensuring reasonable reproducibility of biological replicates. β-tubulin peak area varied by 5–8% within technical replicates (instrument variability) and less than 2-fold within biological replicates (extraction, antennae reproducibility). Average %CV for all peptides was excellent, at <25% (uncorrected peak area) for antennae analysis and comparable for heads/THAs.

#### CircWave cosinor analysis

CircWave v1.4 software (www.huttlab.nl, www.euclock.org, a cosinor analysis program, courtesy of Dr. Roelof Hut,) was used to analyze the rhythmicity of gene and protein levels by fitting a Fourier-curve (one sine wave) to the data^53^. The p values reported are the result of F test from software.

### EAG recordings

Female *An. gambiae* (4 days post emergence, mated but not blood-fed [i.e. host-seeking state]) were immobilized on ice. The head of the mosquito was excised with surgical microscissors (World Precision Instruments, Sarasota, FL) and mounted on an indifferent electrode. Electrodes contained chloridized silver wires in drawn-out glass capillaries filled with 0.1% KCl and 0.5% polyvinylpyrrolidone (PVP). The recording electrode accommodated the two antennae of the excised head after the tips of the antennae were clipped to provide a better contact. Electrode placement (5–10 min) in the dark was made possible by a 16 LED red light source (MaxMax, Carlstadt, NJ; 640 nm peak; 0.35–1.6 mW/in^2^ at the level of the mosquito). Signals were amplified and directly recorded via an IDAC4-USB box (Syntech, Germany). Recordings were analyzed with EAG Pro version 1.1 software (Syntech). The preparation was held in a charcoal filtered and humidified continuous air stream (2.4 liters/min) delivered via a glass tube. A stimulus pulse (1.8 liters/min) was added to the air stream for 0.5 s. To prevent changes in air flow during stimulation, a charcoal-filtered air flow (0.6 liters/min) was delivered via another solenoid valve through a blank syringe into the glass tube and at the same distance from the preparation. Any change in antennal deflection induced by the stimuli or control puffs was recorded for 10 s. Animals tested at ZT12 and ZT16 were exposed to the normal 1 hr dusk dimming cycle. ZT16 group animals were maintained in a dark box (0–1 lux red light) until time of electrophysiological preparation. Red light provided by fluorescent lamps (Philips TLD 36 W/15, 660 nm peak, 4.5 μW/in^2^ at level of mosquito) was used to aid experimental procedures during the dark phase of the LD cycle. In the dark-adapted experiment, the ZT8 group was placed into a dark box at ZT4 and tested in red light at ZT8 (thereby matching the conditions of the ZT16 group).

#### Stimuli

Chemicals of highest purity were used. Indole (98% purity), 3-methylindole (98%), hexanoic acid (98%), geranyl acetone (98%) and *p*-cresol (99%) were from Sigma-Aldrich; nonanal (95%) was purchased from Fluka. Chemicals were dissolved in the solvents dichloromethane (DCM; HPLC grade) or hexane (glass distilled) to make a stock solution of 100 μg/μl and decadic dilutions were made. An aliquot (10 μl) of a stimulus was loaded onto a filter paper strip, the solvent was evaporated for 30 s, and the strip was placed in a 5 ml polypropylene syringe from which various volumes were delivered onto the EAG preparation.

#### Data Analysis

Each recording was first baseline normalized, smoothed with a 10 min running average, amplitude determined and the value of the appropriate blank (solvent) control subtracted. Statistical analyses on the effects of time of day and chemical concentration were performed using SigmaPlot 12 (Systat Software, Chicago, IL) and GraphPad Prism 5 (GraphPad Software, La Jolla, CA). Non-parametric statistical analyses were used when Shapiro–Wilk normality test failed (p < 0.05) or data were square root transformed to correct for non-normal distributions.

### Immunoblot analysis

Three mosquito heads from each time point were homogenized in 1X lysis buffer (30 mM Tris-HCl, pH 6.8, 10% SDS, 0.0002% bromophenol blue, 5% β-mercaptoethanol, 10% glycerol). Lysate from the equivalent of ~1 *An. gambiae* head was loaded and separated on a Novex NuPAGE 4–12% Bis-Tris gel (Life Technologies, Carlsbad, CA) and transferred to a polyvinylidene difluoride membrane. Membranes were blocked and probed overnight with a 1:1000 dilution of rabbit Agam\Orco (Or7, AGAP002560) antiserum^54^. Agam\Orco was detected by horseradish peroxidase-linked goat anti-rabbit IgG (1:2000, GE Healthcare Life Sciences) and developed with the ECL Western Blotting Detection System (GE Healthcare Life Sciences, Piscataway, NJ) per manufacturer's protocol. Two major bands were observed at ~50 kDa (consistent with the predicted molecular weight of 54 kDa) and ~100 kDa, which we hypothesize is an Agam\Orco heterodimer. As a loading control, membranes were stripped and re-probed with a 1:2000 dilution of mouse anti-β-actin mAb JLA 20 (Developmental Studies Hybridoma Bank). Actin was detected by horseradish peroxidase-linked goat anti-mouse IgG (1:2000, GE Healthcare Life Sciences) and developed. Densitometry was conducted using Image J software version 1.42q (NIH, Bethesda, MD).

### Behavioral assays

Individual mosquito locomotor/flight activity was measured with a Locomotor Activity Monitor 25 (LAM 25) system (TriKinetics, Waltham, MA), as previously detailed^3^. Briefly, individual mated but not blood-fed adult female 4–6 days post-emergence (i.e. host-seeking state) mosquitoes were placed in 25 × 150 mm clear glass tubes with access to 20% HFCS in the tubes provided *ad libitum*. Flight activity recorded as infrared beam breaks per minute. All recordings occurred in a light-proof box with its own lighting system in a 12 hr/12 hr LD cycle (11 hr full light, 11 hr darkness (0 lux), and 1 hr dawn and 1 hr dusk transitions) with full light measured at the level of the LAM 25 between 69 and 119 lux. Mosquitoes were monitored for 7 full days. Locomotor flight activity was visualized in actogram format using ClockLab version 2.61 (Actimetrics, Wilmette, IL).

For blood-feeding preference assays, 4–8 days post-emergence mated adult female not blood-fed (i.e. host-seeking state) mosquitoes were separated into individual containers. Blood-feeding began the next morning. Mosquitoes were exposed (within inches) to human arm (the investigator's) for 6 min. Mosquitoes were then anesthetized with CO_2_ and visually assayed for the presence of any blood in the abdomen. To exclude potential human circadian effects, similar assays were performed with mosquitoes raised under reverse LD cycle conditions and then exposed to the human investigator at various phases of the circadian cycle. Similar time-of-day specific results were attained.

## Author Contributions

M.M.C. and J.P.G. performed proteomics experiments. N.A.B. performed and analyzed electrophysiology experiments. M.T.L. performed immunoblot experiments. C.M.H. and N.A.B. analyzed data and prepared figures. S.S.C.R. collected tissue, performed flight activity and blood-feeding experiments, analyzed data and wrote the paper. G.E.D., Z.S. and M.M.C. designed experiments, analyzed data, and wrote the paper.

## Supplementary Material

Supplementary Information Supp Info

Supplementary Information Supplementary Dataset 1

Supplementary Information Supplementary Dataset 2

## Notes

**Accession codes**: Proteomics data have been deposited at the PeptideAtlas: http://www.peptideatlas.org/PASS/PASS00300.
