# Supplementary material for: Daily rhythms in antennal protein and olfactory sensitivity in the malaria mosquito Anopheles gambiae
Source: Sci Rep. 2013 Aug 29;3:2494. doi: 10.1038/srep02494 (PMC3756343; doi:10.1038/srep02494)
Supplement: Supplementary Information — Supp Info [file srep02494-s1.pdf]

Daily rhythms in antennal protein and olfactory sensitivity in the malaria mosquito *Anopheles gambiae*

Supplemental Information

Samuel S.C. Rund, Nicolle A. Bonar, Matthew M. Champion, John P. Ghazi, Cameron M. Houk, Matthew T. Leming, Zainulabeuddin Syed and Giles E. Duffield

Supplementary Dataset 1 Protein ID List & FDR.
Distinct peptide level false discovery rate (FDR) analysis, spectral level FDR analysis, protein level data, distinct peptide level data, spectral level data, single results table, protein summary and peptide summary for proteomics.

Supplementary Dataset 2 Antennae MRM list.
Q1 (m/z), Q3 (m/z), CE (V) and dwell (ms) values for the peptides transitions targeted in this study. For total head appendages, 205 transitions from this list were targeted.

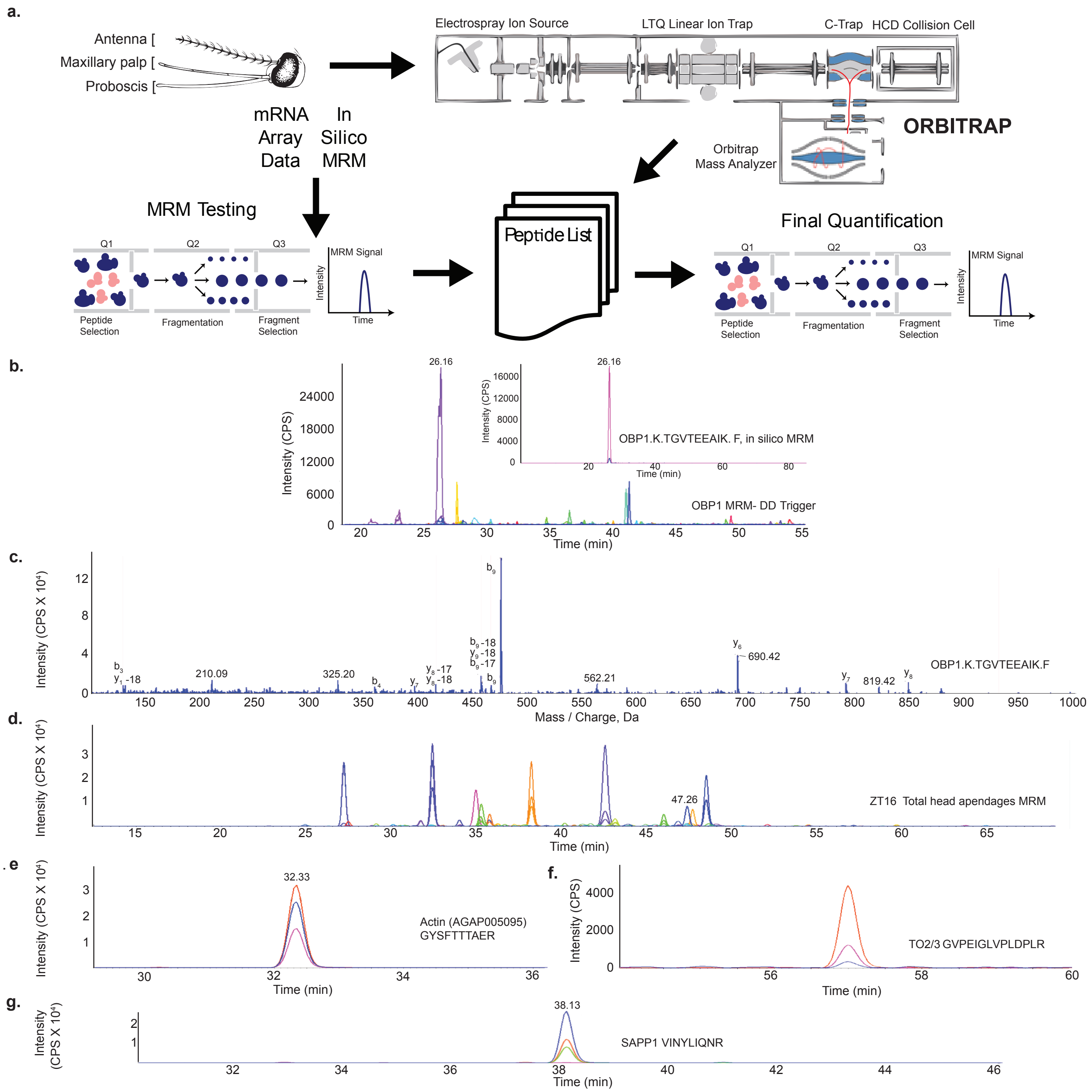
a.
Antenna [
Maxillary palp [
Proboscis [
mRNA Array Data
In Silico MRM
Electrospray Ion Source
LTQ Linear Ion Trap
C-Trap
HCD Collision Cell
ORBITRAP
Orbitrap Mass Analyzer
MRM Testing
Q1
Q2
Q3
Peptide Selection
Fragmentation
Fragment Selection
MRM Signal
Intensity
Time
Peptide List
Final Quantification
b.
Intensity (CPS)
26.16
OBP1.K.TGVTEEAIK. F, in silico MRM
Time (min)
OBP1 MRM- DD Trigger
c.
Intensity (CPS X 10⁴)
b₉
b₉-18
y₉-18
b₉-17
y₆
690.42
b₃
y₁-18
210.09
325.20
b₄
y₇
y₈-17
y₈-18
562.21
y₇
819.42
y₈
OBP1.K.TGVTEEAIK.F
Mass / Charge, Da
d.
Intensity (CPS X 10⁴)
47.26
ZT16 Total head apendages MRM
Time (min)
e
Intensity (CPS X 10⁴)
32.33
Actin (AGAP005095)
GYSFTTTAER
Time (min)
f.
Intensity (CPS)
TO2/3 GVPEIGLVPLDPLR
Time (min)
g.
Intensity (CPS X 10⁴)
38.13
SAPP1 VINYLIQNR
Time (min)

Figure S1 Targeted proteomics method development.

(a) Steps taken to develop the time-of-day specific collection of MRM transitions from *An. gambiae*. Briefly, bottom-up data dependent acquisition was used with retention time (RT) filtering to determine the majority of the peptides to monitor. Peptides were selected based on previously published mRNA-based data that revealed 24 hr gene expression patterns of OBPs and other related transcripts, and identified and confirmed with MRM-data dependent acquisition. The curated peptide list was analyzed across time point replicates to determine rhythmic proteins. Modified Orbitrap image from Thermo Fisher Scientific. **(b)** Example analysis from an *in silico* MRM determined transitions with the output from predicted peptides of OBP1 (AGAP003309); TIC (total ion current) for all MRM transitions detected in the run. *Inset*, a single OBP1 transition pulled out for sequencing. **(c)** Annotated spectrum of K.TGVTEEAIK.F (M+H)2+ 474.3 m/z. Highlighted are all matched b and y type ions, the transitions at 690.4y6, 789.4y7, and 589.3y5 were used in subsequent MRM analysis. **(d)** Representative ion chromatogram of all monitored peptide transitions within the MRM analysis (205 transitions shown) from mosquito-extracted proteins at the ZT16 time point. Data here are from extracted total head appendages. **(e)** Typical high abundance MRM signal (predicted actin, AGAP005095) [GYSFTTTAER]. **(f)** A typical lower abundance MRM signal (TO2/3, AGAP012703/AGAP004262) [GVPEIGLVPLDPLR]. Shown here are three confirmatory transitions, the most intense (red) is used for quantification, the remaining transitions (two in this case) are qualifiers. (H) Peptide from SAPP1 (AGAP008051) [VINYLIQNR] that has an identical mass (60mmu) to the predicted actin peptide in panel E, illustrating the specificity of this approach.

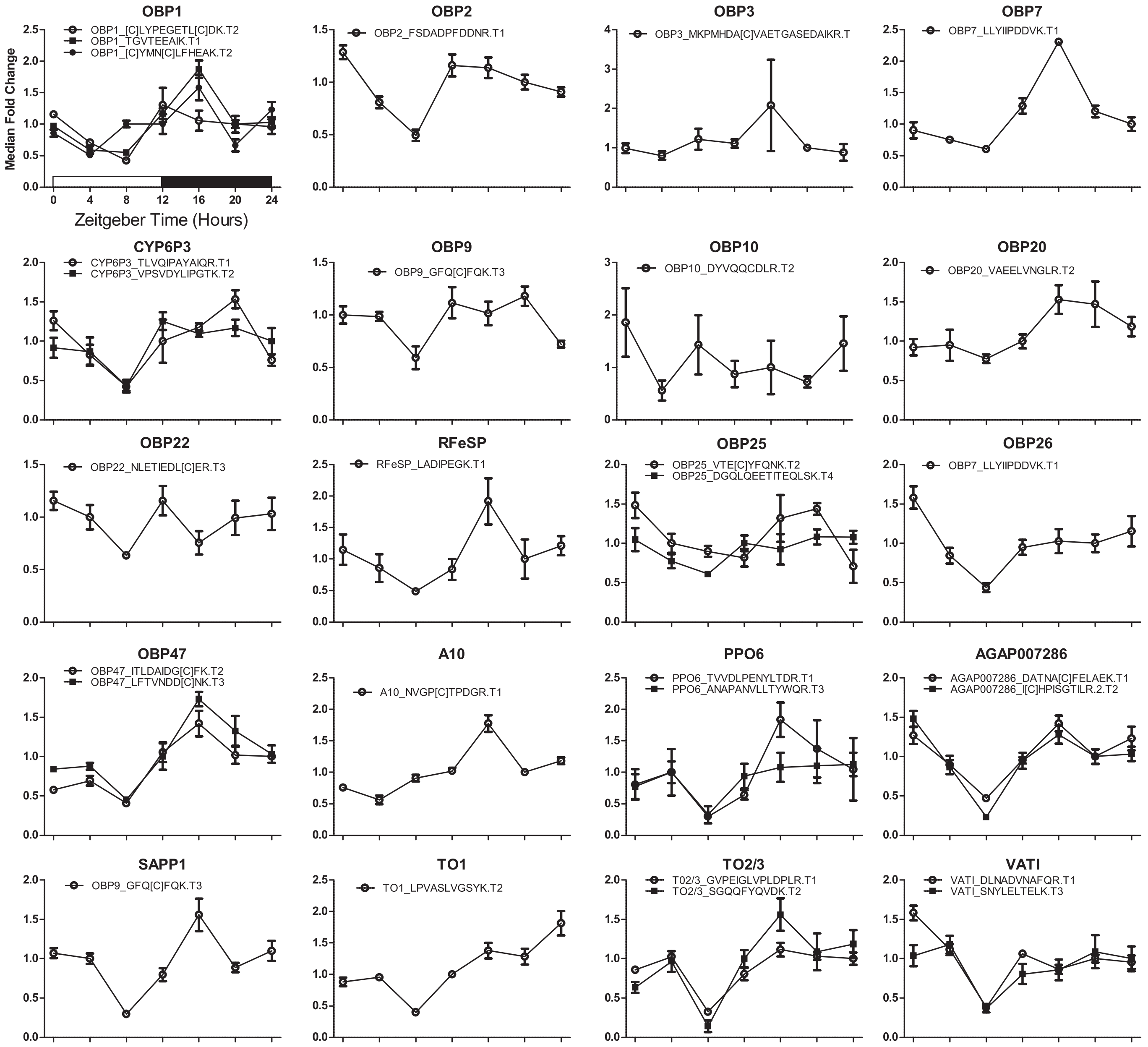
OBP1
OBP1_[C]LYPEGETL[C]DK.T2
OBP1_TGVTEEAIK.T1
OBP1_[C]YMN[C]LFHEAK.T2
Median Fold Change
Zeitgeber Time (Hours)
OBP2
OBP2_FSDADPFDDNR.T1
OBP3
OBP3_MKPMHDA[C]VAETGASEDAIKR.T
OBP7
OBP7_LLYIIPDDVK.T1
CYP6P3
CYP6P3_TLVQIPAYAIQR.T1
CYP6P3_VPSVDYLIPGTK.T2
OBP9
OBP9_GFQ[C]FQK.T3
OBP10
OBP10_DYVQQCDLR.T2
OBP20
OBP20_VAEELVNGLR.T2
OBP22
OBP22_NLETIEDL[C]ER.T3
RFeSP
RFeSP_LADIPEGK.T1
OBP25
OBP25_VTE[C]YFQNK.T2
OBP25_DGQLQEETITEQLSK.T4
OBP26
OBP7_LLYIIPDDVK.T1
OBP47
OBP47_ITLDAIDG[C]FK.T2
OBP47_LFTVNDD[C]NK.T3
A10
A10_NVGP[C]TPDGR.T1
PPO6
PPO6_TVVDLPENYLTDR.T1
PPO6_ANAPANVLLTYWQR.T3
AGAP007286
AGAP007286_DATNA[C]FELAEK.T1
AGAP007286_I[C]HPISGTILR.2.T2
SAPP1
OBP9_GFQ[C]FQK.T3
TO1
TO1_LPVASLVGSYK.T2
TO2/3
T02/3_GVPEIGLVPLDPLR.T1
TO2/3_SGQQFYQVDK.T2
VATI
VATI_DLNADVNAFQR.T1
VATI_SNYLELTELK.T3

Figure S2 Individual quantifier peptide identities and levels.

Final quantification of every protein was averaged from the median transformed expression of 1-3 individual component peptides. Here we show the individual quantifier peptide identities and levels (mean ± S.E.M.) prior to averaging the peptides from the first biological replicate antennae time course. Peptide profiles shown were normalized to tubulin peptide signal. For this figure, all gene symbols are listed as in VectorBase except for the following: RFeSP (homologue to *Drosophila* Rieske iron-sulfur protein, AGAP008955), A10 (homologue to *Drosophila* antennal protein 10, AGAP008055), VATI (predicted V-type proton ATPase catalytic subunit I, AGAP001587) and AGAP007286 (*Ae. aegypti* OBP43 homologue) and IDH (predicted isocitrate dehydrogenase, AGAP006660).

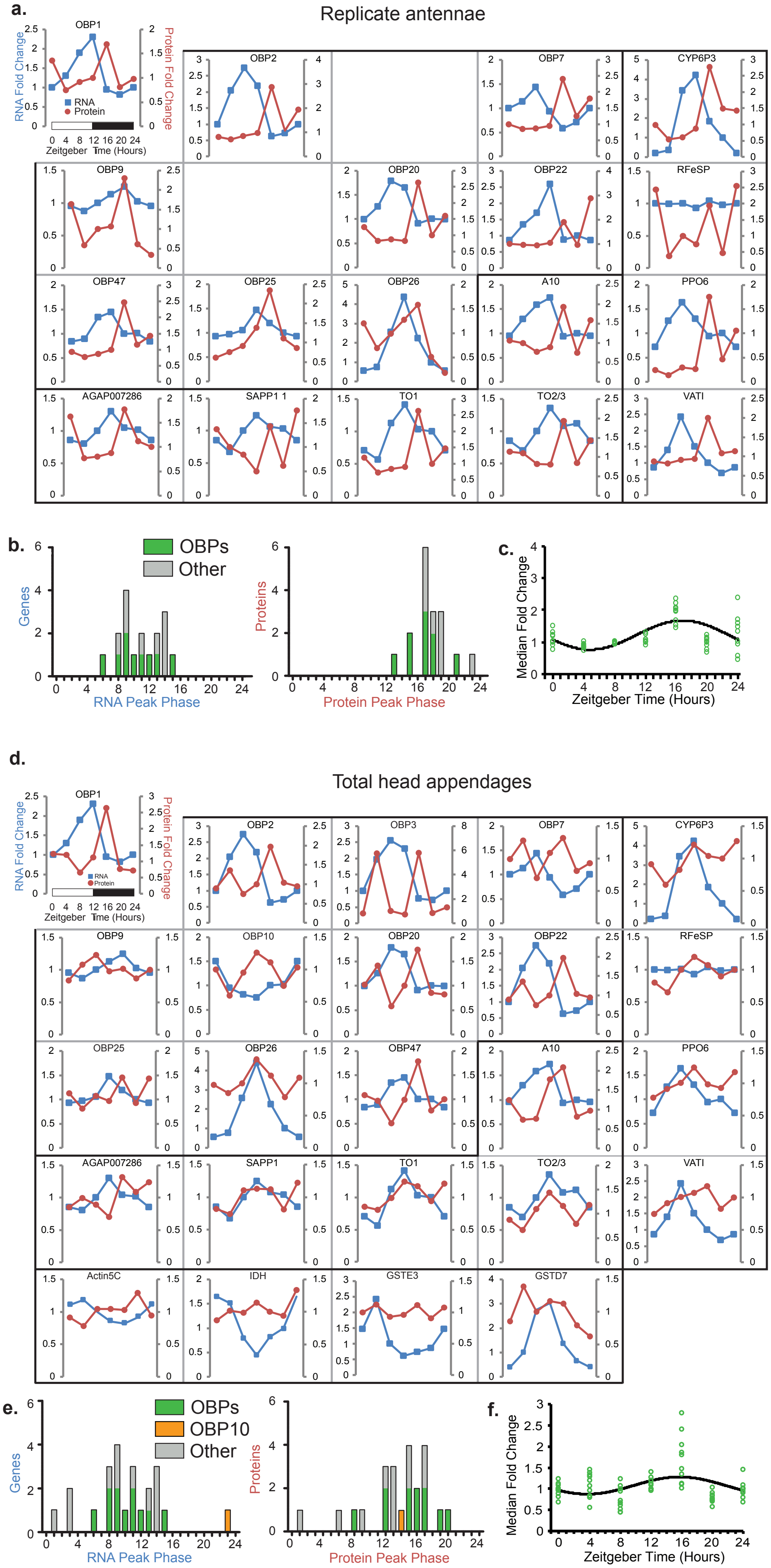

a.
Replicate antennae
OBP1
RNA Fold Change
Protein Fold Change
RNA
Protein
Zeitgeber Time (Hours)
OBP2
OBP7
CYP6P3
OBP9
OBP20
OBP22
RFeSP
OBP47
OBP25
OBP26
A10
PPO6
AGAP007286
SAPP1 1
TO1
TO2/3
VATI
b.
OBPs
Other
Genes
RNA Peak Phase
Proteins
Protein Peak Phase
c.
Median Fold Change
Zeitgeber Time (Hours)
d.
Total head appendages
OBP1
RNA Fold Change
Protein Fold Change
RNA
Protein
Zeitgeber Time (Hours)
OBP2
OBP3
OBP7
CYP6P3
OBP9
OBP10
OBP20
OBP22
RFeSP
OBP25
OBP26
OBP47
A10
PPO6
AGAP007286
SAPP1
TO1
TO2/3
VATI
Actin5C
IDH
GSTE3
GSTD7
e.
OBPs
OBP10
Other
Genes
RNA Peak Phase
Proteins
Protein Peak Phase
f.
Median Fold Change
Zeitgeber Time (Hours)

Figure S3 Protein rhythms from antennae and total head appendages (THAs).

(a) Targeted quantitative proteomics of antennae from a second biological replicate time course confirms rhythms in mosquito antennal protein correspond highly with RNA expression profiles determined from whole-head samples. Mosquitoes were collected every 4 hr for 24 hr, antennae collected and targeted quantitative proteomics performed. Proteins are grouped into OBPs, non-OBP chemosensory proteins (AGAP007286, SAPP1, A10, TO1 and TO2/3) and non-olfactory proteins (CYP6P3, RFeSP, PPO6 and VATI). Protein abundance normalized with tubulin. See Fig. 2 for protein rhythms in the first antennae replicate time course. **(b)** Histogram of peak RNA expression phases in total heads compared to their peak antennal protein levels as determined by cosinor analysis from genes/proteins in panel A. RFeSP excluded as it is not rhythmic at the RNA level. **(c)** Cosinor analysis of antennal OBP protein levels from panel A ($p < 0.001$; acrophase ZT16.8). **(d)** Targeted quantitative proteomics on THAs correspond highly with RNA expression profiles determined from whole-head samples. Note the bottom row of non-olfactory proteins (Actin5c, IDH, GSTE3 and GSTD7) were subjected to targeted proteomics in THAs but not fully evaluated in the antennae. **(e)** Histograms of genes with peak RNA expression phases in total heads compared with the corresponding peak THAs protein levels (determined by cosinor analysis from genes/proteins in panel D, RFeSP excluded). **(f)** Cosinor analysis of THAs OBP protein levels, OBP3 excluded as it has an atypical double peak in protein levels ($p < 0.01$; acrophase ZT14.9).

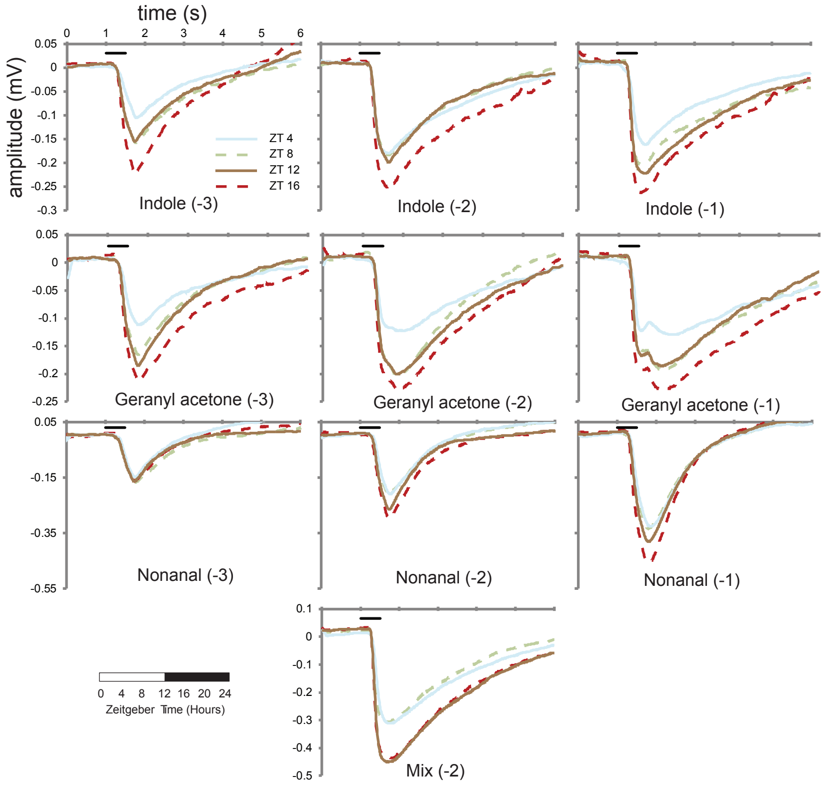

Figure S4 Dose-dependent olfactory responses from *An. gambiae* antennae to major host derived chemostimuli.

For each stimulus (top to bottom), the mean trace at each of the four times of day tested and the three tested doses (left to right) are presented. The black bar at 1.0 s represents the onset of 0.5 s stimulus delivery. Note that these traces are not blank normalized.
